# Crossmodal Statistical Binding of Temporal Information and Stimuli Properties Recalibrates Perception of Visual Apparent Motion

**DOI:** 10.3389/fpsyg.2016.00434

**Published:** 2016-03-29

**Authors:** Yi Zhang, Lihan Chen

**Affiliations:** ^1^Department of Psychology and Beijing Key Laboratory of Behavior and Mental Health, Peking UniversityBeijing, China; ^2^Key Laboratory of Machine Perception (Ministry of Education), Peking UniversityBeijing, China

**Keywords:** Ternus display, temporal structure, intersensory binding, statistical learning, interval

## Abstract

Recent studies of brain plasticity that pertain to time perception have shown that fast training of temporal discrimination in one modality, for example, the auditory modality, can improve performance of temporal discrimination in another modality, such as the visual modality. We here examined whether the perception of visual Ternus motion could be recalibrated through fast crossmodal statistical binding of temporal information and stimuli properties binding. We conducted two experiments, composed of three sessions each: pre-test, learning, and post-test. In both the pre-test and the post-test, participants classified the Ternus display as either “element motion” or “group motion.” For the training session in Experiment 1, we constructed two types of temporal structures, in which two consecutively presented sound beeps were dominantly (80%) flanked by one leading visual Ternus frame and by one lagging visual Ternus frame (VAAV) or dominantly inserted by two Ternus visual frames (AVVA). Participants were required to respond which interval (auditory vs. visual) was longer. In Experiment 2, we presented only a single auditory–visual pair but with similar temporal configurations as in Experiment 1, and asked participants to perform an audio–visual temporal order judgment. The results of these two experiments support that statistical binding of temporal information and stimuli properties can quickly and selectively recalibrate the sensitivity of perceiving visual motion, according to the protocols of the specific bindings.

## Introduction

In a typical temporal ventriloquism effect, perception of the onset of a visual event or the intervals of paired visual events is biased by the presentation of nearby auditory clicks or paired auditory beeps (Chen and Vroomen, 2013). For example, Morein-Zamir et al. (2003) showed that when presenting a sound before the first light and a second sound after the second light (the AVVA configuration), participants could more easily differentiate the two lights, as if the sounds pulled the lights further apart in time. In contrast, when the two sounds occurred in between the two lights, the sounds apparently pulled the lights closer together and made it difficult to judge the order of visual lights, rendering participants’ performance less accurate (Morein-Zamir et al., 2003). The temporal ventriloquism effect has recently been extended to dynamic scenarios by employing the visual Ternus display (Shi et al., 2010). The Ternus display involves a multi-element stimulus that can induce either of two different percepts of apparent motion: “element motion” or “group motion.” In this study, each frame had two disks, with the second disk of the first frame and the first disk of the second frame being presented at the same location. The perception of “element motion” or “group motion” is dependent on the perceived interval between the two Ternus frames. When the inter-frame interval is short, observers perceive “element motion,” in which the endmost disk is seen as moving back and forth while the middle disk, at the central position, remains stationary or flashing. When the inter-frame interval is longer, observers generally perceive “group motion,” in which both disks appear to move laterally as a whole. The two perceptions are mutually exclusive. The visual Ternus display thus provides a good tool for manipulating crossmodal temporal disparities. This study also found that two sounds presented in temporal proximity to, or synchronously with, the two visual frames, respectively, can shift the transitional threshold for visual apparent motion. However, such effects were not evident with single-sound configurations (Shi et al., 2010).

Temporal perception bias has been demonstrated not just in the one trial demonstration of audiovisual integration, but also after an adaptation procedure. Here, the auditory and visual events each occur separately beyond the time window in which multisensory integration could have taken place (Spence and Squire, 2003). Using a temporal adaptation task and employing the Ternus apparent motion as probes, Zhang et al. (2012) found that adapting to different time intervals conveyed through stimuli in different modalities affects the subsequent implicit perception of visual timing. The stimuli in different modalities could be frames of a visual Ternus display, visual blinking disks, or auditory beeps. Adapting to the short time interval in all of the above situations led to more reports of “group motion” for the subsequent Ternus display. However, adapting to the long time interval gave rise to different results. In this condition, no aftereffects for visual adaptation occurred, while there were significantly more reports of group motion for auditory adaptation (Zhang et al., 2012). Additionally, Chen and Zhou (2014), also using the Ternus apparent motion as probes, examined the extent to which the ability to discriminate sub-second time intervals acquired in one sensory modality can be transferred to another modality with a fast perceptual training protocol. The training protocol required participants to explicitly compare the interval length between a pair of visual, auditory, or tactile stimuli with a standard interval. Results showed that after fast explicit training of interval discrimination (about 15 min), participants improved their ability to categorize the visual apparent motion in the Ternus displays. However, the training benefits here were mild for visual timing. Overall, in light of the evidence of crossmodal transfer of time perception and adaptation, it seems a central clock may account for sub-second temporal processing (Ivry and Schlerf, 2008; Chen and Zhou, 2014).

Beyond temporal manipulations, previous studies have investigated the role of feature binding in crossmodal time perception. Evidence so far supports that a single auditory event can selectively bind with only one of multiple visual events, or alternatively, interact with all of the visual events, to reach a perceptual decision (simultaneity judgment or feature discrimination) on them (Van der Burg et al., 2008, 2013; Roseboom et al., 2009, 2013). This flexible association of temporal pairings is also shown in a person’s own actions and sensory feedback. In this case, exposing the left and right hands to different action-effect lags can concurrently lead to different amounts of the temporal recalibration effect (Sugano et al., 2014).

The different and selective adaptation reported in the above studies has indeed addressed the aftereffects of fixed temporal relations between different sensory events or between the action and its feedback. The current study asks whether perception of time intervals in one modality can be implicitly biased by inferring temporal relations between crossmodal events, in which the observers should use both the temporal information and stimuli properties. The statistical binding of temporal information and stimuli properties, implemented through presentations of probable audiovisual events, would let the observers form a temporary prior assessment of the temporal (interval) relations between the target events. Hence, the observable temporal aftereffects would be rendered. Moreover, statistical binding of temporal information and stimuli properties could largely form strong temporal perceptual groupings, which would otherwise be less obvious or absent with single or fewer trials of the presentation of audiovisual pairs (see Experiment 2 in Shi et al., 2010). In the present study, we investigated this hypothesis by constructing selective temporal relations between visual Ternus frames (with black or red elements) and auditory beeps. We expected that the temporal interval modulations between the paired auditory beeps and the visual Ternus frames would give rise to different adaptation aftereffects. This would then lead to different biases of perceiving “element motion” vs. “group motion” in the post-test of the Ternus display. We conducted two experiments, detailed below, to examine our hypotheses.

## Materials and Methods

The procedure of pre-test, training, and post-test was adopted. The pre-test and post-test tasks were discriminations of visual Ternus apparent motion (“element motion” vs. “group motion”). The interim training sessions were tasks of temporal discrimination of auditory–visual events– either interval comparison (Experiment 1) or temporal order judgment (TOJ; Experiment 2).

## Experiment 1

In Experiment 1, we manipulated the temporal interval structure between paired auditory–visual events. We set up two configurations of the Ternus display. That is, the Ternus frame contained either two black disks or two red disks. We paired mostly (80% of total trials) the black frames with a temporal structure in which two visual Ternus frames were inserted between two auditory beeps (the VAAV configuration). Meanwhile, two red frames were mainly associated with another temporal structure– two beeps were inserted between two visual Ternus frames (the AVVA configuration). We hypothesized that the statistically dominant VAAV configuration would lead to a decrease in sensitivity for visual intervals, and this influence would generalize to the Ternus motion [with increased just noticeable differences (JNDs)]. In contrast, the dominant AVVA structure would lead to an increase in sensitivity for visual intervals, decreasing the JNDs for judging Ternus motion in the post-test.

### Participants

Twenty-eight students (15 females) from Peking University took part in Experiment 1. The mean age of the sample was 22.1 years old. Seventeen students (nine females) attended Experiment 1a, in which the sample had a mean age of 21.9 years old. Eleven students (six females) participated in Experiment 1b, in which the sample had a mean age of 22.2 years old. All the participants had normal or corrected-to-normal vision, and normal hearing. All were naïve as to the purpose of the experiment. The study was approved by the Ethics Committee of the Department of Psychology at Peking University and informed consent was obtained before the experiment for all participants.

### Stimuli and Apparatus

**FIGURE 1:**
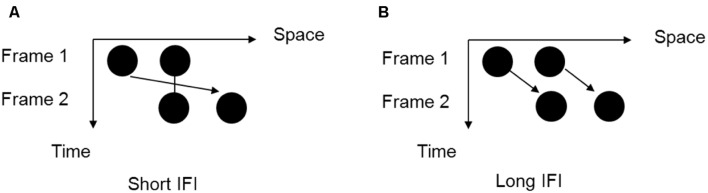
**The two possible motion perceptions of the Ternus display. (A)** “Element motion” (with a short inter-frame interval): the center dot is perceived to remain at the same spot, while the outer dot is perceived to move from one side to the other side. **(B)** “Group motion” (with a long inter-frame interval): the two dots are perceived to move together as a group.

The visual stimuli consisted of two frames, each containing two disks (1.3° of visual angle in diameter) presented on a gray background (16.1 cd/m^2^ luminance). The disks were either red (10.6 cd/m^2^ luminance) or black (12.7 cd/m^2^ luminance) and the disks in each trial were of the same color. The separation between the two disks was 2° of visual angle. As shown in **Figure 1**, the two frames shared one element location at the center of the monitor but contained two other elements located at horizontally opposite positions relative to the center.

Visual stimuli were presented on a 22-inch CRT monitor (1,024 × 768 pixels; 100 Hz) controlled by a PC (HPAMD Athlon 64 Dual-Core Processor) with a Radeon 1700 FSC graphics card. Viewing distance was set to 57 cm, maintained by using a chinrest. The testing room was dimly lit with an average ambient luminance of 0.12 cd/m^2^. Audio stimuli (65 dB, 1,000 Hz) were generated and delivered via an M-Audio card (Delta 1010) to a headset (RT-788V, RAPTOXX). Stimulus presentation and data collection were implemented by computer programs which were developed with Matlab 7.1 (MathWorks Inc., Natick, MA, USA) and Psychophysics Toolbox (Brainard, 1997; Pelli, 1997).

### Design and Procedure

A between-participants design was adopted in Experiment 1. Experiment 1a was composed of three sections: pre-test, training, and post-test. In the pre-test and post-test, a Ternus display showing either two black frames or two red frames was used. In Experiment 1b (the control test for Experiment 1a), the pre-test and post-test were the same as in Experiment 1a, except that the participants were required to take a rest during the time equivalent to that of the training session in Experiment 1a.

#### Pre-test and Post-test

Before the formal experiment, participants underwent practice to become familiar with a Ternus display of both the typical element motion (ISI = 50 ms) and group motion (ISI = 200 ms) percepts. They were asked to discriminate the above two percepts by pressing the left and right mouse button to indicate judgments of each element motion and group motion, respectively. The mapping between button and response type was counterbalanced across participants. When participants made an incorrect response, immediate feedback appeared on the screen showing the percept (element motion or group motion) that they should have reported. The practice session continued until the participant’s report accuracy was close to 100%. Almost all of the participants met this standard within 120 trials.

In the pre-test phase, each trial began with a fixation cross presented at the center of the display for 300 ms, followed by a blank display with a random interval of 500–700 ms. Next, typical Ternus motion was depicted as two frames with a random ISI (50, 80, 110, 140, 170, 200, or 230 ms) as shown in **Figure 1**. Each Ternus frame was presented for 30 ms. After another blank display for 500 ms, a question mark appeared to prompt participants to make a forced-choice response by using the mouse button. The next trial began 500 ms after the participant pressed the button. There were 24 trials for each ISI level. Color (red or black) and the directions of apparent motion (leftward or rightward) were balanced across trials. The 336 trials were divided into four blocks and participants could take a short rest between blocks. There was no feedback in the pre-test session.

The procedure of the post-test phase was the same as that of the pre-test phase.

#### Training

**FIGURE 2:**
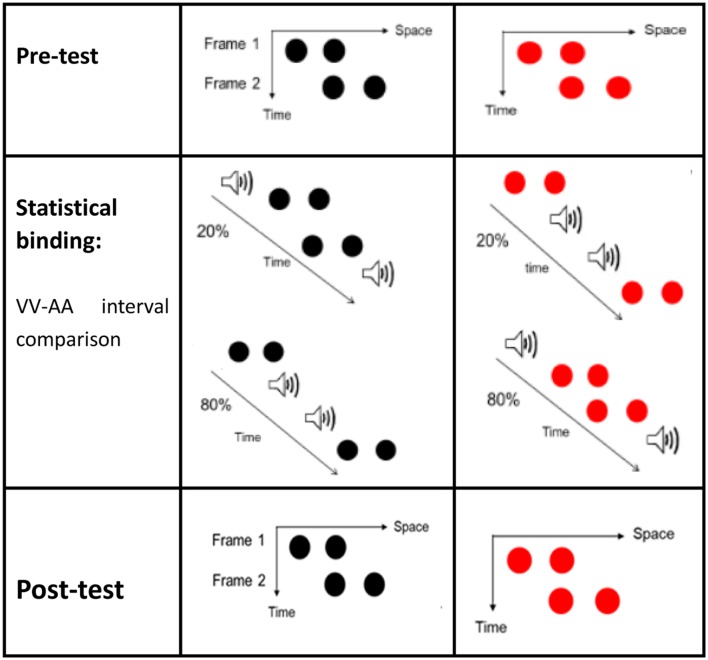
**Ternus displays (for the pre-test and post-test) and illustrations of the stimuli configurations for Experiment 1.** Two kinds of Ternus displays were used (with black frames or red frames). In the training session, for 80% of the red–red (RR) configuration trials, the first sound preceded the first red visual frame and the second sound trailed the second red visual frame by 80 ms (hereafter referred to simply as the AVVA condition). In 80% of the black–black (BB) configuration trials, the first visual frame preceded the first beep and the second visual frame trailed the second beep by 80 ms (hereafter referred to simply as the VAAV condition). The inter-stimulus-interval (ISI) between the two Ternus frames was randomly set from between 50 and 230 ms inclusive. For the other 20% of the trials, the RR and BB configurations were associated with temporal structures of VAAV and AVVA, respectively.

Participants in the training group were required to complete an interim training session on temporal interval discriminations before the post-test. After participants saw a fixation cross for 300 ms and a blank display for 300–500 ms, two frames would appear on the screen with a random ISI of 50–230 ms. Each frame contained two disks presented consecutively at the center of the screen. The color of the disks in a given trial was either black or red. Two brief 30 ms sound beeps appeared along with the two visual stimuli. There were two conditions of the audiovisual interval. In condition 1, for 80% of the trials, the first sound preceded the first red visual frame and the second sound trailed the second red visual frame by 80 ms (the AVVA condition). **Figure 2** shows that in condition 2, for 80% of the trials, the first sound trailed the first black visual Ternus frame by 80 ms and the second sound preceded the second black visual frame by 80 ms. These were called “inner sounds” (VAAV temporal structure). For the less common condition (20% of trials), the temporal structures of AVVA and VAAV were used in reverse to those in the more common condition (80% trials). When the audiovisual stimuli were presented again, after another blank screen of 500–700 ms, text appeared on the screen asking “Which time interval is longer, the visual or the sound beep?” Participants were then prompted to respond by pressing the associated mouse key (the left key to indicate that the auditory interval was longer and the right key to indicate that the visual interval was longer). When they were incorrect, immediate feedback would appear on the screen telling them so. The next trial began 1–1.2 s after the participant pressed the button. A total of 360 trials was divided into six blocks between which participants could take a short rest.

### Results

#### Pre-test and post-test

**FIGURE 3:**
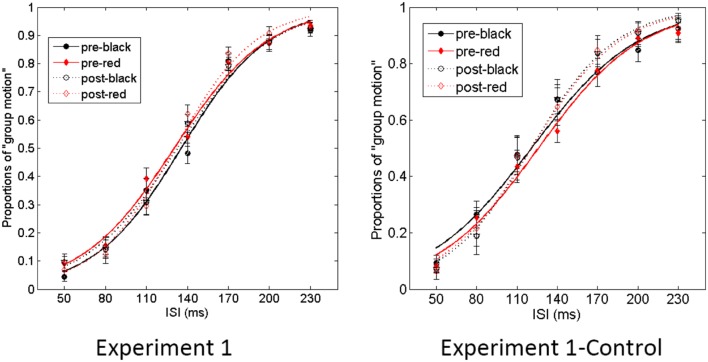
**Average psychometric curves for Experiment 1.** In the graph “Experiment1” **(Left)**, the solid line with solid black circles shows the percentage of “group motion” for the pre-test of the black Ternus display. The solid line with solid red diamonds indicates the pre-test of the red Ternus display. The dotted line with empty circles indicates the percentage of “group motion” for the post-test of the black Ternus display, and the dotted line with empty diamonds the post-test of the red Ternus display. In the graph “Experiment 1-Control” **(Right)**, the various colored curves indicate the same as in the left graph. The error bars in both graphs represent the SEM.

**FIGURE 4:**
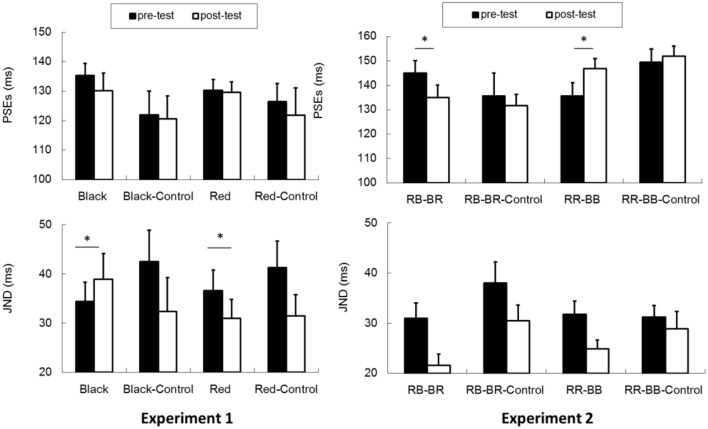
**PSEs and JNDs for Experiment 1 and Experiment 2.** Experiment 1: “Black” (Experimental Condition for the black Ternus frames), “Black–Control” (Control Condition for the black Ternus frames), “Red” (Experimental Condition for the red frames of the Ternus display), “Red–Control” (Control Condition for the red Ternus frames). Experiment 2: “RB–BR”: Consecutively presented two visual frames of red–black and black–red disks. “RR–BB”: Consecutively presented two visual frames of red–red and black–black disks. The error bars represent standard errors. An asterisk (^∗^) indicates a significant effect (*p* < 0.05).

The proportion of group motion reports was plotted as a function of ISI and fit by a logistic regression for each participant (**Figure 3**). For each condition (black vs. red Ternus frame), the transitional threshold between element motion and group motion, that is, the point at which group motion and element motion were reported with equal frequency, was calculated by estimating the 50% performance point on the (fitted) logistic function. The transitional threshold is also referred to as the point of subjective equality (PSE). The just noticeable difference (JND) represents the difference between the two motion perceptions, which is obtained by estimating the ISI difference of half between 25% and 75% of the group motion responses from the psychometric curves (Treutwein and Strasburger, 1999). PSEs were 135.3 ± 4.1 (SE) and 130.3 ± 3.7 for the pre-test of the black Ternus type and the pre-test of the red Ternus type, respectively. PSEs were 130.1 ± 6.1 and 129.6 ± 3.6 for the post-test of the black Ternus type and the post-test of the red Ternus type, respectively. A repeated measures analysis of variance (ANOVA) with independent factors [Ternus type: Color (black or red) vs. Time (pre-test or post-test)] showed that the PSEs were statistically equal for the black Ternus and red Ternus, *F*(1,16) = 1.355, *p* = 0.262. There was no significant difference between PSEs across pre-test and post-test, *F*(1,16) = 0.841, *p* = 0.373. No interaction between the two factors was found either, *F*(1,16) = 1.272, *p* = 0.276. These results are shown in **Figure 4**.

The JNDs were 34.4 ± 3.9 (SE) and 36.6 ± 4.2 for the pre-test of the black Ternus type and the pre-test of the red Ternus type. JNDs were 38.9 ± 5.2 and 31.0 ± 3.8 for the post-test of the black Ternus type and the post-test of the red Ternus type. A repeated measures ANOVA with independent factors [Ternus type: Color (black or red) vs. Time (pre-test or post-test)] showed that the JNDs were statistically equal for the black Ternus and the red Ternus, *F*(1,16) = 1.783, *p* = 0.200. There was no significant difference between JNDs across the pre-test and post-test, *F*(1,16) = 0.105, *p* = 0.750. Importantly, however, the interaction between the factor of Ternus type and Time was significant, *F*(1,16) = 10.034, *p* < 0.01. Simple main effects analysis showed that after training with the VAAV temporal structure, the JNDs for the black visual Ternus motion were increased, *F*(1,16) = 5.32, *p* < 0.05. In contrast, after training with the AVVA temporal structure, the JNDs for the red visual Ternus motion were decreased, *F*(1,16) = 4.67, *p* < 0.05.

Used as a control for Experiments 1a,b showed PSEs of 121.9 ± 8.1 (SE) and 126.4 ± 6.2 for the pre-test of the black Ternus type and the pre-test of the red Ternus type. PSEs were 120.6 ± 7.8 and 121.8 ± 9.3 for the post-test of the black Ternus type and the post-test of the red Ternus type. A repeated measures ANOVA with independent factors [Ternus type: Color (black or red) vs. Time (pre-test or post-test)] showed that the PSEs were statistically equal for the black Ternus and the red Ternus, *F*(1,10) = 1.017, *p* = 0.337. There was no significant difference between PSEs across the pre-test and post-test, *F*(1,10) = 0.538, *p* = 0.480. The interaction between the factors of Ternus type and Time was also not significant, *F*(1,10) = 0.288, *p* = 0.603.

For Experiment 1b, the JNDs were 42.5 ± 6.4 (standard error) and 41.3 ± 5.4 for the pre-test of the black Ternus type and pre-test of the red Ternus type. The JNDs were 32.4 ± 6.8 and 31.5 ± 4.3 for the post-test of black Ternus type and post-test of the red Ternus type. A repeated measures ANOVA with independent factors (Ternus type: Color (black or red) vs. Time (pre-test or post-test) showed that the JNDs were statistically equal for the black Ternus and the red Ternus, *F*(1,10) = 0.090, *p* = 0.770. However, the JNDs were significantly reduced across the pre-test and post-test, *F*(1,10) = 7.554, *p* < 0.05. The interaction between the factor of Ternus type and Time was not significant, *F*(1,10) = 0.006, *p* = 0.938.

Overall, Experiments 1a and 1b showed that without auditory–visual temporal interval training, the post-test of Ternus motion increased the sensitivity of categorizing between element motion vs. group motion, and that this improvement was probably due to increased familiarity with the task in the post-test. Importantly though, the different statistical training on the VAAV and AVVA temporal structures led to opposite aftereffects (changes in JNDs) on the perception of Ternus apparent motion: the VAAV condition led to decreased sensitivities for Ternus motion perception while the AVVA condition led to the opposite- sharpened sensitivities for Ternus motion perception.

#### Training Performance

The mean accuracy of discrimination between auditory intervals and visual intervals for the VAAV temporal structures was 89.6% (2.7%). The mean accuracy for the AVVA temporal structures was 86.6% (3.6%). Thus, the accuracy rate in the VAAV condition was higher than the one in the AVVA condition, *t*(16) = 3.071, *p* < 0.01. However, both accuracy rates were above the chance level of 50%, *p*s < 0.001. The results indicate that the training task was successful.

## Experiment 2

In Experiment 2, we broke up the paired visual and auditory events and presented only a single auditory–visual pair in each trial. The training task was audio–visual TOJ. We examined whether and how the intersensory binding of temporal orders of auditory and visual events bias responses in the post-test of Ternus motion. Although a single sound has not been shown to be potent enough to influence visual apparent motion (Bruns and Getzmann, 2008; Shi et al., 2010), we hypothesized that through intersensory binding, perceptual grouping of dominant temporal structures could still occur, and be used to influence the subsequent perception of visual Ternus motion.

### Participants

Fifty-eight students (35 females) from Peking University took part in Experiment 2. The mean age of the sample was 21.6 years old. The participants were separated into four groups and each group performed only one of the following experimental tasks. For Experiment 2a, there were 15 participants (12 females) with a mean age of 20.4 years old. This group received the “RB–BR” Ternus configuration with interim training on TOJ. Experiment 2b constituted the control group for Experiment 2a. In Experiment 2b, there were 11 participants (six females) with a mean age of 22.4 years old. These participants received the “RB–BR” Ternus configuration without training. In Experiment 2c there were 17 participants (nine females) whose mean age was 21.2 years old. These participants received the “RR–BB” Ternus configuration with interim training on TOJ. Experiment 2d constituted the control group for Experiment 2c. In Experiment 2d there were 15 participants (eight females) whose mean age was 21.3 years old. These participants received the “RR–BB” Ternus configuration without training.

All participants had normal or corrected-to-normal vision and normal hearing. All were naïve as to the purpose of the experiment. The study was approved by the Ethics Committee of the Department of Psychology at Peking University and informed consent was obtained before the experiment for all participants.

### Stimuli and Apparatus

Stimuli and apparatus were the same as those in Experiment 1, except that the color of the disks was different. Details will be described in the following sections.

### Design and Procedure

Experiment 2 was a 2 (type of Ternus configuration: element motion vs. group motion) × 2 (test group: experiment vs. control) factorial between-participants design.

In Experiment 2, we used a variant of the Ternus paradigm. The direction of the Ternus apparent motion was always from left to right in Experiment 2. We composed the typical dominant element motion and the typical group motion percepts based on the color feature bindings of the Ternus component disks (Kramer and Yantis, 1997; Petersik and Rice, 2008). In the typical element motion condition, in each frame and from left to right, the first Ternus frame contained red and black disks, while the second Ternus frame was composed of black and red disks (referred to as “RB” and “BR” frames). This configuration gave rise to the dominant percept of element motion. In the typical group motion configuration, the first frame was composed of two red disks while the second frame was composed of two black disks (referred to as “RR” and “BB” frames).

#### Pre-test and Post-test

In Experiment 2, the settings of the demo and practice, as well as the procedure of the pre-test and post-test were the same as those in Experiment 1, except that the apparent motion direction of the Ternus display was always from left to right. For Experiments 2a and 2b, participants were required to discriminate between element motion and group motion based on the RB–BR Ternus configuration. For Experiments 2c and 2d, participants were asked to discriminate between element motion and group motion based on the RR–BB Ternus display. In both Ternus settings, the ISI between the two visual frames was from 50 to 230 ms (with 30 ms as a step size).

#### Training

In the training phase, participants performed a TOJ task. After the appearance of a fixation cross (for 300 ms) and a blank display (for 300–500 ms), a visual frame as well as a sound beep appeared with a random stimulus onset asynchrony (SOA) of 50–150 ms. When the presentation of audiovisual stimuli was over, participants were prompted to indicate which stimulus came first. Half of the group was required to press the left mouse key if they perceived the beep first, and the right key if the visual frame was first. The other half of the group reversed the mapping between the response and the stimuli. The inter-trial interval (ITI) was 800–1000 ms. Upon each incorrect response, the word “Wrong” appeared in red on the center of the screen, to give accuracy feedback to the participants. There were 360 trails in total and the training session was separated into six blocks. Participants were asked to take a rest between blocks.

**FIGURE 5:**
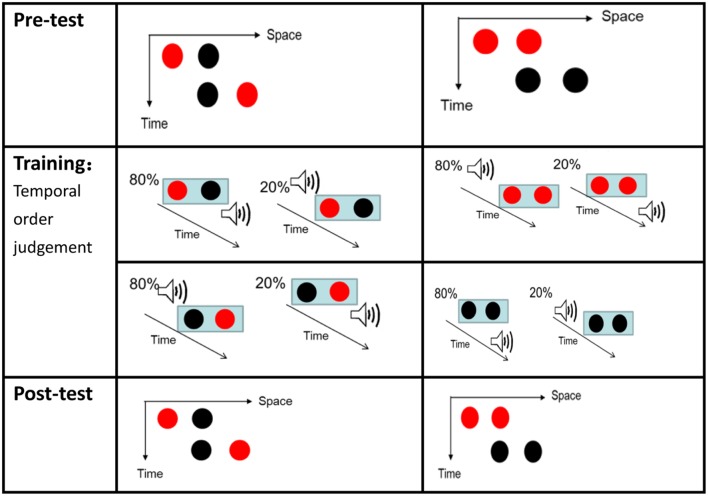
**Ternus displays (for the pre-test and post-test) and illustrations of the stimuli configurations for Experiment 2.** Two kinds of Ternus displays were used in Experiment 2. The middle of the left side of the figure depicts the training session with the Red–Black Black–Red (RB–BR) configuration. In 80% of the trials here, the Red–Black frame preceded the sound beep and the Black–Red frame trailed the beep. In contrast, in 20% of these trials, the Red–Black frame trailed the sound beep and the Black–Red frame preceded the sound beep. The middle of the right side of the figure depicts the Red–Red Black–Black (RR–BB) frame configuration. In 80% of these trials, the Red–Red frame trailed the sound beep and the Black–Black frame preceded the sound beep. In contrast, in 20% of these trials, the Red–Red frame preceded the sound beep and the Black–Black frame trailed the sound beep.

The temporal disparities between auditory–visual pairs and the statistical distribution of those disparities were as follows. In the RB–BR configuration (i.e., the typical element motion percept), 80% of the RB frames preceded the sound beep and 80% of the BR frames trailed the beep. In contrast, 20% of RB frames trailed the sound beep and 20% of the BR frames preceded the sound beep. Henceforth the above distribution would lead to a subjectively dominant perceptual grouping of VAAV. In the RR–BB configuration (typical group motion percept), 80% of the RR frames trailed the sound beep and 80% of the BB frames preceded the beep. In contrast, 20% of RR frames preceded the sound beep and 20% of the BB frames trailed the sound beep. Henceforth the above distribution would lead to a subjectively dominant perceptual grouping of AVVA. This pattern is shown in **Figure 5**.

### Result

We first conducted a one-way ANOVA of the PSEs and JNDs in the pre-test across the four experiments. The results were as follows: for the PSEs, *F*(3,57) = 1.284, *p* = 0.289. For the JNDs, *F*(3,57) = 1.071, *p* = 0.369. These results indicate that the baselines for the performance on the Ternus motion tasks between the experimental group and control group were comparable. Therefore, we carried out an independent analysis for each sub-experiment.

**FIGURE 6:**
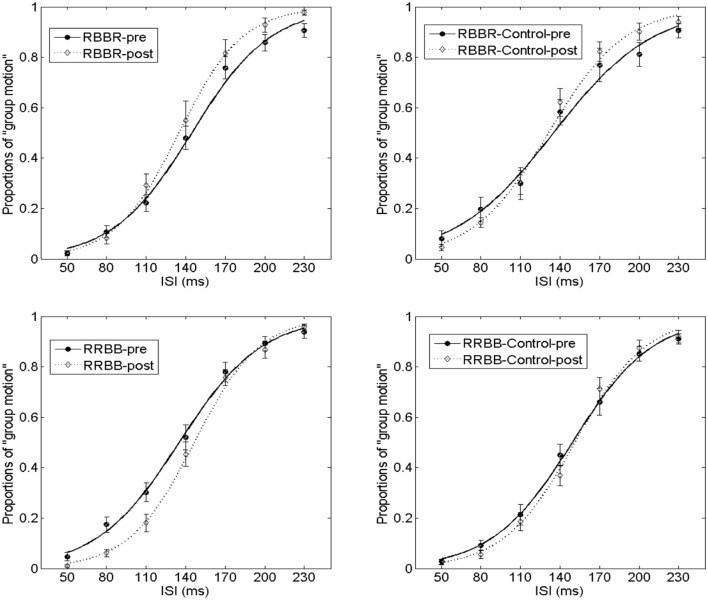
**Average psychometric curves for Experiment 2 and its control.** In the RBBR configuration, the solid line with filled-black circles shows the percentage of group motion for the pre-test of the RBBR Ternus display, and the dotted line with empty diamonds indicates the percentage of group motion for the post-test of RB–BR. With the RRBB configuration, the connotations of the line and associated markers are the same as in the RBBR condition. For RBBR the visual Ternus display contained Red–Black and Black–Red frames (from left to right). For RRBB the visual Ternus display contained Red–Red and Black–Black frames (from left to right). The error bars represent SEM.

In Experiment 2a (the element motion configuration), the PSEs were 145.0 ± 5.1 and 134.9 ± 5.1 for the pre-test and post-test, *t*(14) = 2.574, *p* < 0.05. The JNDs were 31.0 ± 3.0 and 21.6 ± 2.2 for the pre-test and post-test, *t*(14) = 3.112, *p* < 0.01. In Experiment 2b (the element motion control condition), the PSEs were 135.6 ± 9.4 and 131.6 ± 4.7 for the pre-test and post-test, *t*(10) = 0.507, *p* = 0.623. The JNDs were 38.0 ± 4.2 and 30.5 ± 3.1 for the pre-test and post-test, *t*(10) = 2.692, *p* < 0.05. Therefore, the training and intersensory binding VAAV temporal structure led to a decreased PSE and more dominant perception of group motion. This is shown in **Figure 6**.

In Experiment 2c (the group motion configuration), the PSEs were 135.6 ± 5.5 and 146.8 ± 4.1 for the pre-test and post-test, *t*(16) = –2.578, *p* < 0.05. The JNDs were 31.8 ± 2.6 and 24.9 ± 1.7 for the pre-test and post-test, *t*(16) = 4.834, *p* < 0.001. In Experiment 2d (the group motion control condition), the PSEs were 149.5 ± 5.5 and 151.9 ± 4.1 for the pre-test and post-test, *t*(14) = -0.792, *p* = 0.442. The JNDs were 31.2 ± 2.3 and 28.9 ± 3.4 for the pre-test and post-test, *t*(14) = 0.905, *p* = 0.381. Therefore, the training and intersensory binding AVVA temporal structure led to an increased PSE and more dominant perception of element motion.

For the training sessions in both experiments, the accuracy of reporting temporal order was better than the chance level. The correct rate was 95.2 ± 1.2% for the AV TOJ training in Experiment 2a and 95.6 ± 0.8% in Experiment 2c. Therefore, the performance of TOJ was satisfactory.

## Discussion

The present study used variants of visual Ternus displays to examine whether fast crossmodal statistical binding of temporal information and sensory properties would recalibrate and hence bias the perception of visual Ternus motion (element motion vs. group motion). This was indexed by the changes in the PSEs and JNDs of the post-test Ternus apparent motion, compared with the pre-test ones. To achieve this, we manipulated the pairing of audiovisual events in the training session, so that one temporal structure would be statistically dominant over the other. The training tasks were either temporal interval comparisons (Experiment 1) or TOJs (Experiment 2). Therefore, the aftereffects of the training were mainly due to crossmodal statistical binding of temporal information and stimuli properties in the training session.

### Intersensory Binding of Temporal Structure and Audiovisual Properties

In Experiment 1, we created the VAAV temporal structure by putting two black Ternus frames outside of one pair of auditory beeps 80% of the time, so that in this case, the visual interval was longer than the auditory interval. We composed the AVVA temporal structure by putting two red Ternus frames temporally inside the paired auditory beeps 80% of the time, so that the visual interval was shorter than the auditory intervals. According to the temporal precision hypothesis, the auditory interval should calibrate the visual interval (Burr et al., 2009; Shi et al., 2010). With the VAAV condition, the inserted paired beeps would pull the two black visual frames closer in time, leading to increased JNDs (lower sensitivities) for judging Ternus motion in the post-test. Using the same reasoning, in the AVVA configuration, the flanked (outside) two beeps would pull the two red Ternus frames further away in time, leading to a subjectively extended interval between the two visual frames, and hence higher sensitivities for discriminating Ternus motion. Indeed, from the obtained JNDs, we observed increased sensitivities (smaller JNDs) for Ternus motion in the AVVA condition and decreased sensitivities (larger JNDs) for Ternus motion in the VAAV condition. Compared with the null changes of the PSEs and JNDs in the control tests (only the pre-test and post-test of Ternus apparent motion), the results from the training protocol suggest that the intersensory binding of the temporal structure as well as audiovisual properties had generalized to affect perception of the implicit ‘interval’ between the two visual frames in the Ternus display. This led to the perceptual decision of element motion versus group motion.

### Intersensory Binding across Space, Time, and Experimental Trials

One might argue that in Experiment 1 the symmetric alignments of paired audiovisual events on both ends could potentially induce a response bias. This response bias might cause participants to make TOJs solely on the time asynchrony cues of either the first audiovisual pair or the second audiovisual pair, due to ignorance of the temporal intervals between auditory and visual events. To rule out this possibility, and to provide a comparison for the manipulation in Experiment 1, we directly examined the intersensory binding of visual property and auditory signal when the audiovisual pair was always presented alone. This ensured it would be task-demanding for participants to form the potential interval comparisons between visual pair and auditory pair through the combinations of the onsets and offsets of the two consecutively presented single audiovisual pairs. This was in contrast to Experiment 1, in which two audio visual pairs were always concurrently present, so that intersensory binding between visual property (color) and the auditory beeps (and hence the temporal intervals) occurred spontaneously and was less task-demanding.

Note that in Shi et al. (2010), the authors showed that the transitional threshold for visual apparent motion can be shifted only when the two sounds are presented in temporal proximity to the two visual frames. Such effects were not evident with single-sound configurations. This result suggests that two sounds are required to induce the temporal ventriloquism effect. In other words, the sounds influence the perceived time interval rather than the onset or offset time of visual events. In Experiment 2 of the present study, we explored the main factor that influences visual time perception: the onset and offset time, or, the time interval of the auditory stimuli. If the effect occurs because of the onset and offset time of the auditory stimuli, learning the time information associated with a single pair of asynchronous audiovisual stimuli would be enough to induce the temporal ventriloquism aftereffect. We would further expect to observe more perceptions of element motion for the post-test of the RB–BR Ternus configuration, due to the fact that the two inside auditory beeps would draw the RB and BR visual frame closer. By the same token, the post-test of the RR–BB Ternus display should lead to more reports of group motion. However, this was not the case. In the absence of the events just described, we would expect that the interval information (with both onsets and offsets) is responsible for the temporal recalibration effect. Indeed, we found that in Experiment 2, in which intersensory binding occurred. Observers had formed the dominant temporal structures of V(RB)-AA-V(BR) and A-V(RR)V(BB)-A through the inherent intersensory binding between visual frames of different colors, and their spatial locations, as well as the temporal relations with respect to the corresponding sound beeps. They exploited the different audiovisual interval information to calibrate the probe-test of Ternus apparent motion.

### Mechanisms of ‘Lag Adaptation’ vs. ‘Bayesian Calibration’

As observed in Experiment 2, a “positive” interval adaptation aftereffect contributed to the differing results obtained: (1) with the VAAV temporal structure, the interval between paired visual frames was subjectively extended and led to more reports of group motion (with reduced PSEs) in the post-test; (2) with the AVVA structure, the interval between the paired visual frames was contracted subjectively and led to more reports of element motion (with increased PSEs) This replicated the results of Zhang et al. (2012). Both temporal structures have led to increased sensitivities of discriminating Ternus motion in the post-test. This positive aftereffect was analogous to the “lag adaptation” revealed in Fujisaki et al. (2004) and other studies, in which they reported that after exposure to a fixed audiovisual time lag for several minutes, human participants showed shifts in their subjective simultaneity responses toward that particular lag (Heron et al., 2007, 2012; Hanson et al., 2008; Harrar and Harris, 2008; Roseboom and Arnold, 2011; Machulla et al., 2012).

In contrast to the findings of Experiment 2, the results from Experiment 1 showed a pattern of the Bayesian calibration (negative) aftereffects (Miyazaki et al., 2006; Yamamoto et al., 2012). In Bayesian negative calibration, when the temporal asynchronies between crossmodal events were sampled from a prior probability distribution (usually a Gaussian distribution), exposure to the above asynchronies led to opposite perceptual changes, and conformed to predictions derived from Bayesian integration theory. In our case, the exposure of subjectively extended “Black–Black” Ternus frame intervals led rather to increased JNDs, and the opposite occurred for “Red–Red” Ternus frames in the post-tests. We inferred that the Bayesian calibration was always at work in both Experiment 1 and Experiment 2. Lag adaptation is advantageous for adjusting variable sound delays that exist in the real world and has been shown to operate in (single) audiovisual pair integration. Thus we speculate that in Experiment 2, the lag adaptation mechanism counteracted the shift of PSEs due to Bayesian calibration, particularly in the judgment of audiovisual intervals, and played an upper hand in determining the temporal aftereffects. This mode of competition between “Bayesian calibration” and “lag adaptation” has also been shown in Yamamoto et al. (2012).

### Implicit Statistical Binding of Temporal Information and Stimuli Properties

Statistical learning, as a theoretical construct, was offered as a general mechanism for learning and processing any type of sensory input that unfolds across time and space (Frost et al., 2014). The present study applied different levels of statistical learning and indicated domain-general ability as well as stimulus-specific constraints in the learning. The learning here refers to updating the internal temporal (interval) representation of the given crossmodal input and encoding potential temporal relations between them. In this way, improvement occurs in the processing of that input and transfers to the post-test of implicit time perception. Here, it manifests as implicit perception of the time interval in the Ternus display. This approach to learning has been investigated explicitly in Roseboom and Arnold (2011). They showed that humans can form multiple concurrent estimates of appropriate timing for audiovisual synchrony, and that audiovisual temporal recalibration can be specific for particular audiovisual pairings. Specifically, participants in Roseboom and Arnold (2011) were shown alternating movies of male and female actors containing positive and negative temporal asynchronies between the auditory and visual streams. The authors found that audiovisual synchrony estimates for each actor were shifted toward the preceding audiovisual timing relationship for that actor and that such temporal recalibration occurred in positive and negative directions concurrently (Roseboom and Arnold, 2011). Here we found that in addition to forming fixed temporal asynchrony between audiovisual pairings, this intersensory binding could be exploited implicitly by exposure to the more frequent pairing of audiovisual events, but with random temporal asynchronies and correspondence of sensory properties. The intersensory binding in our case may happen more automatically since in the “learning” session, the Ternus frames themselves received less attention than the temporal relations between visual Ternus and auditory beeps. By using an implicit time perception paradigm (Ternus display), the present study has shown a new type of temporal recalibration as a result of comprehensive intersensory binding across time, space, and other sensory properties (such as visual color).

Despite the potential contributions just described, we acknowledge several limitations in the current study. There is an underlying assumption that sound can influence the time perception of visual events because of greater temporal precision with auditory events. However, we did not explore the potential influence of visual events upon auditory time perception in general, nor when the auditory information is blurred. A direction for future research is to explore whether, with appropriate auditory probes, visual temporal information will dominate auditory information in calibrating the auditory timing task. And although we established the competitive advantages of the Bayesian calibration aftereffects (Experiment 1) and Lag-like adaptation aftereffects (Experiment 2), we did not explore how the specifics of their dominance affect the interpretation of the post-tests and underlying neural mechanisms. This too, is a worthy endeavor for future research.

In summary, by using the typical Ternus effect paradigm, the present study examined crossmodal binding between visual and auditory events across the properties of space, color, and time. The results indicated that depending on the specific binding protocols, statistical binding of temporal information and stimuli properties can concurrently and selectively recalibrate the implicit-time perception of visual intervals. Thus, this binding can influence the perceived states of visual apparent motion.

## Author Contributions

LC and YZ designed the experiments. YZ conducted the experiments. LC and YZ analyzed the data and wrote the manuscript.

## Conflict of Interest Statement

The authors declare that the research was conducted in the absence of any commercial or financial relationships that could be construed as a potential conflict of interest.

## References

[B1] Brainard D. H. (1997). The psychophysics toolbox. *Spat. Vis.* 10 433–436. 10.1163/156856897X00357 9176952

[B2] BrunsP.GetzmannS. (2008). Audiovisual influences on the perception of visual apparent motion: exploring the effect of a single sound 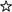 . *Acta Psychol. (Amst)* 129 273–283. 10.1016/j.actpsy.2008.08.00218790468

[B3] Burr D. Banks M. S. Morrone M. C. (2009). Auditory dominance over vision in the perception of interval duration. *Exp. Brain Res.* 198 49–57. 10.1007/s00221-009-1933-z 19597804

[B4] Chen L. Vroomen J. (2013). Intersensory binding across space and time: a tutorial review. *Atten. Percept. Psychophys.* 75 790–811. 10.3758/s13414-013-0475-4 23709064

[B5] Chen L. Zhou X. (2014). Fast transfer of crossmodal time interval training. *Exp. Brain Res.* 232 1855–1864. 10.1007/s00221-014-3877-1 24570386

[B6] Frost R. Armstrong B. C. Siegelman N. Christiansen M. H. (2014). Domain generality versus modality specificity: the paradox of statistical learning. *Trends Cogn. Sci.* 19 117–125. 10.1016/j.tics.2014.12.010 25631249 PMC4348214

[B7] Fujisaki W. Shimojo S. Kashino M. Nishida S. (2004). Recalibration of audiovisual simultaneity. *Nat. Neurosci.* 7 773–778. 10.1038/nn1268 15195098

[B8] Hanson J. V. Heron J. Whitaker D. (2008). Recalibration of perceived time across sensory modalities. *Exp. Brain Res.* 185 347–352. 10.1007/s00221-008-1282-3 18236035

[B9] Harrar V. Harris L. R. (2008). The effect of exposure to asynchronous audio, visual, and tactile stimulus combinations on the perception of simultaneity. *Exp. Brain Res.* 186 517–524. 10.1007/s00221-007-1253-0 18183377

[B10] Heron J. Roach N. W. Hanson J. V. McGraw P. V. Whitaker D. (2012). Audiovisual time perception is spatially specific. *Exp. Brain Res.* 218 477–485. 10.1007/s00221-012-3038-3 22367399 PMC3324684

[B11] Heron J. Whitaker D. McGraw P. V. Horoshenkov K. V. (2007). Adaptation minimizes distance-related audiovisual delays. *J. Vis.* 7 5 1–5.8. 17997633 10.1167/7.13.5

[B12] Ivry R. B. Schlerf J. E. (2008). Dedicated and intrinsic models of time perception. *Trends Cogn. Sci.* 12 273–280. 10.1016/j.tics.2008.04.002 18539519 PMC4335014

[B13] Kramer P. Yantis S. (1997). Perceptual grouping in space and time: evidence from the Ternus display. *Percept. Psychophys.* 59 87–99. 10.3758/BF03206851 9038411

[B14] Machulla T. K. Di Luca M. Froehlich E. Ernst M. O. (2012). Multisensory simultaneity recalibration: storage of the aftereffect in the absence of counterevidence. *Exp. Brain Res.* 217 89–97. 10.1007/s00221-011-2976-5 22207361

[B15] Miyazaki M. Yamamoto S. Uchida S. Kitazawa S. (2006). Bayesian calibration of simultaneity in tactile temporal order judgment. *Nat. Neurosci.* 9 875–877. 10.1038/nn1712 16732276

[B16] Morein-Zamir S. Soto-Faraco S. Kingstone A. (2003). Auditory capture of vision: examining temporal ventriloquism. *Brain Res. Cogn. Brain Res.* 17 154–163. 10.1016/S0926-6410(03)00089-2 12763201

[B17] Pelli D. G. (1997). The VideoToolbox software for visual psychophysics: transforming numbers into movies. *Spat. Vis.* 10 437–442. 10.1163/156856897X00366 9176953

[B18] Petersik J. T. Rice C. M. (2008). Spatial correspondence and relation correspondence: grouping factors that influence perception of the Ternus display. *Perception* 37 725–739. 10.1068/p5900 18605146

[B19] Roseboom W. Arnold D. H. (2011). Twice upon a time: multiple concurrent temporal recalibrations of audiovisual speech. *Psychol. Sci.* 22 872–877. 10.1177/0956797611413293 21690312

[B20] Roseboom W. Kawabe T. Nishida S. (2013). The cross-modal double flash illusion depends on featural similarity between cross-modal inducers. *Sci. Rep.* 3:3437 10.1038/srep03437 PMC3853685 24310546

[B21] Roseboom W. Nishida S. Arnold D. H. (2009). The sliding window of audio-visual simultaneity. *J. Vis.* 9 1–8. 10.1167/9.12.4 20053095

[B22] Shi Z. Chen L. Muller H. J. (2010). Auditory temporal modulation of the visual Ternus effect: the influence of time interval. *Exp. Brain Res.* 203 723–735. 10.1007/s00221-010-2286-3 20473749

[B23] Spence C. Squire S. (2003). Multisensory integration: maintaining the perception of synchrony. *Curr. Biol.* 13 R519–R521. 10.1016/S0960-9822(03)00445-7 12842029

[B24] Sugano Y. Keetels M. Vroomen J. (2014). Concurrent sensorimotor temporal recalibration to different lags for the left and right hand. *Front. Psychol.* 5:140 10.3389/fpsyg.2014.00140 PMC3934310 24624098

[B25] Treutwein B. Strasburger H. (1999). Fitting the psychometric function. *Percept. Psychophys.* 61 87–106. 10.3758/BF03211951 10070202

[B26] Van der Burg E. Awh E. Olivers C. N. (2013). The capacity of audiovisual integration is limited to one item. *Psychol. Sci.* 24 345–351. 10.1177/0956797612452865 23389426 PMC4476285

[B27] Van der Burg E. Olivers C. N. Bronkhorst A. W. Theeuwes J. (2008). Pip and pop: nonspatial auditory signals improve spatial visual search. *J. Exp. Psychol. Hum. Percept. Perform.* 34 1053–1065. 10.1037/0096-1523.34.5.1053 18823194

[B28] Yamamoto S. Miyazaki M. Iwano T. Kitazawa S. (2012). Bayesian calibration of simultaneity in audiovisual temporal order judgments. *PLoS ONE* 7:e40379 10.1371/journal.pone.0040379 PMC3392227 22792297

[B29] Zhang H. Chen L. Zhou X. (2012). Adaptation to visual or auditory time intervals modulates the perception of visual apparent motion. *Front. Integr. Neurosci.* 6:100 10.3389/fnint.2012.00100 PMC3488759 23133408

